# Defining myocardial fibrosis in haemodialysis patients with non-contrast cardiac magnetic resonance

**DOI:** 10.1186/s12872-018-0885-2

**Published:** 2018-07-13

**Authors:** M. P. Graham-Brown, A. S. Singh, G. S. Gulsin, E. Levelt, J. A. Arnold, D. J. Stensel, J. O. Burton, G. P. McCann

**Affiliations:** 1 John Walls Renal Unit, University Hospitals Leicester NHS Trust, Leicester, UK; 2 Department of Infection Immunity and Inflammation, School of Medicine and Biological Sciences, University of Leicester, Leicester, LE1 9HN UK; 3 National Centre for Sport and Exercise Medicine, University of Loughborough, Loughborough, UK; 4 Deparment of Cardiovascular Sciences, University of Leicester and NIHR Leicester Cardiovascular Biomedical Research Centre, Glenfield Hospital Leicester, Leicester, UK

**Keywords:** Haemodialysis, Aortic stenosis, Myocardial fibrosis, Native T1, LGE

## Abstract

**Background:**

Extent of myocardial fibrosis (MF) determined using late gadolinium enhanced (LGE) predicts outcomes, but gadolinium is contraindicated in advanced renal disease. We assessed the ability of native T1-mapping to identify and quantify MF in aortic stenosis patients (AS) as a model for use in haemodialysis patients.

**Methods:**

We compared the ability to identify areas of replacement-MF using native T1-mapping to LGE in 25 AS patients at 3 T. We assessed agreement between extent of MF defined by LGE full-width-half-maximum (FWHM) and the LGE 3-standard-deviations (3SD) in AS patients and nine T1 thresholding-techniques, with thresholds set 2-to-9 standard-deviations above normal-range (1083 ± 33 ms). A further technique was tested that set an individual T1-threshold for each patient (T11SD). The technique that agreed most strongly with FWHM or 3SD in AS patients was used to compare extent of MF between AS (*n* = 25) and haemodialysis patients (n = 25).

**Results:**

Twenty-six areas of enhancement were identified on LGE images, with 25 corresponding areas of discretely increased native T1 signal identified on T1 maps. Global T1 was higher in haemodialysis than AS patients (1279 ms ± 5.8 vs 1143 ms ± 12.49, *P* < 0.01). No signal-threshold technique derived from standard-deviations above normal-range associated with FWHM or 3SD. T11SD correlated with FWHM in AS patients (*r* = 0.55) with moderate agreement (ICC = 0.64), (but not with 3SD). Extent of MF defined by T11SD was higher in haemodialysis vs AS patients (21.92% ± 1 vs 18.24% ± 1.4, *P* = 0.038), as was T1 in regions-of-interest defined as scar (1390 ± 8.7 vs 1276 ms ± 20.5, *P* < 0.01). There was no difference in the relative difference between remote myocardium and regions defined as scar, between groups (111.4 ms ± 7.6 vs 133.2 ms ± 17.5, *P* = 0.26).

**Conclusions:**

Areas of MF are identifiable on native T1 maps, but absolute thresholds to define extent of MF could not be determined. Histological studies are needed to assess the ability of native-T1 signal-thresholding techniques to define extent of MF in haemodialysis patients.

Data is taken from the PRIMID-AS (NCT01658345) and CYCLE-HD studies (ISRCTN11299707).

## Background

The development of myocardial fibrosis (MF) is common to many cardiac pathologies and is associated with poor outcomes [1, 2]. The pathogenesis of MF differs across disease states, but in diseases of pressure or volume overload, patients initially develop diffuse interstitial myocardial fibrosis (DMF) [3]. Over time, increasing levels of DMF lead to decreased capillary density and myocardial ischaemia, with subsequent myocyte apoptosis and development of replacement fibrosis (scar) [4]. Extent of MF is strongly related to adverse outcomes in a number of conditions [5, 6]. Patients with chronic kidney disease (CKD) and end stage renal disease (ESRD) are known to have high levels of MF that are unrelated to coronary artery disease that starts as DMF and progresses with stage of CKD, being most severe in patients on dialysis [7]. Percentage area of LV fibrosis has been shown to be a stronger predictor of cardiovascular outcomes than extent of myocyte hypertrophy in haemodialysis patients and an independent predictor of mortality [8]. The ability to non-invasively detect and define extent of MF in patients with advanced renal disease has significant implications [9, 10].

The assessment of MF with late gadolinium enhanced (LGE) cardiac magnetic resonance imaging (CMR) is well established and validated against histology in experimental models [11–13]. LGE accurately defines areas of replacement fibrosis due to the increased extracellular volume and prolonged washout of contrast related to decreased capillary density within fibrotic myocardial tissue [14, 15]. The presence and extent of scarring defined by LGE is strongly related to adverse outcomes in a number of conditions, independent of cardiac volumes and ejection fraction [1, 5, 6]. Quantifying extent of MF is possible using LGE signal intensity (SI) thresholding techniques such as full-width-half maximum (FWHM) [16], or the 3-standard deviation (3SD) technique which has recently been shown to correlate with extent of MF in aortic stenosis (AS) patients [17]. The reproducibility of these techniques are excellent, but tend to underestimate total fibrosis burden [18, 19], due to the dependence of the technique in demonstrating a difference between signal intensity of normal and fibrotic myocardial tissue [20]. Administration of gadolinium based contrast agents are not possible in patients with advanced renal disease and those on dialysis due to the risk of nephrogenic systemic fibrosis [21], precluding qualitative or quantitative assessment of MF.

The potential for native T1 mapping to quantify DMF has been shown in a wide range of diseases [22–27], including patients with CKD and patients on haemodialysis [28–30]. The generation of native T1 parametric maps allows not only qualitative assessment of myocardium on colour coded parametric maps, but pixel-wise quantification of T1 values [30, 31]. The technique correlates well with levels of MF on histology in AS patients [22] and defining abnormal areas of myocardium is not solely reliant on demonstrating a difference in SI to an area of normal myocardium as in LGE [32]. Whether combined visual and quantitative threshold analysis of parametric native T1 maps can reliably identify and quantify extent of MF is not known. Aortic stenosis is a disease of pressure overload, which shares several morphological myocardial characteristics with uraemic cardiomyopathy, including development of left ventricular hypertrophy, DMF and replacement fibrosis. Comparing LGE images to native T1 images in AS patients serves as a useful model to explore the possibility of using native T1 mapping to identify replacement MF and extent of MF in patients unable to receive gadolinium based contrast agents.

We hypothesised that it is possible to visually identify and quantify areas of replacement fibrosis in ESRD using native T1 mapping.

## Methods

Patients with AS were recruited from the PRIMID-AS study (NCT01658345) and haemodialysis patients were recruited from the CYCLE-HD study (ISRCTN11299707). The design and rationale for both studies are as previously described [33, 34]. Both studies received ethical approval from the National Research Ethics Service Committee East Midlands (REC references 11/EM/0410 and 14/EM/1190, respectively). All participants gave written and informed consent. The scans of 126 patients from the PRIMID-AS study (from host centre, Leicester, UK) and the scans of the first 44 patients to enter the CYCLE-HD study were assessed included.

### Image acquisition

Patients from the PRIMID-AS and the CYCLE-HD studies were scanned on the same 3 T MRI platform (Skyra, Siemens Medical Imaging, Erlangen, Germany) using an 18-channel phased-array anterior coil. CMR protocols for acquisition of LGE images (AS only) and native T1 maps are as previously described [30, 35]. Briefly, pre-contrast short-axis native T1 maps were acquired at the mid-ventricular level using the modified look-locker inversion recovery (MOLLI) sequence. Images were acquired using free-breathing with motion correction (MOCO), ECG-gated single-shot MOLLI sequence [36] with 3(3)3(3)5 sampling pattern and the following typical parameters for both studies: slice thickness 8.0 mm, field of view 300 × 400 mm, flip angle 50 degrees (PRIMID-AS cohort) and 35 (CYCLE-HD cohort), minimum TI 120 ms, inversion-time increment 80 ms. Patients from the PRIMID-AS study underwent a comprehensive adenosine stress and rest perfusion study that included left-ventricular (LV) LGE imaging 7–10 min after administration of gadolinium gadopentate (Gadovist, Bayer, Faversham, United Kingdom) 0.15 mmol/kg.

### Visual comparison of LGE images and native T1 maps

**Fig. 1:**
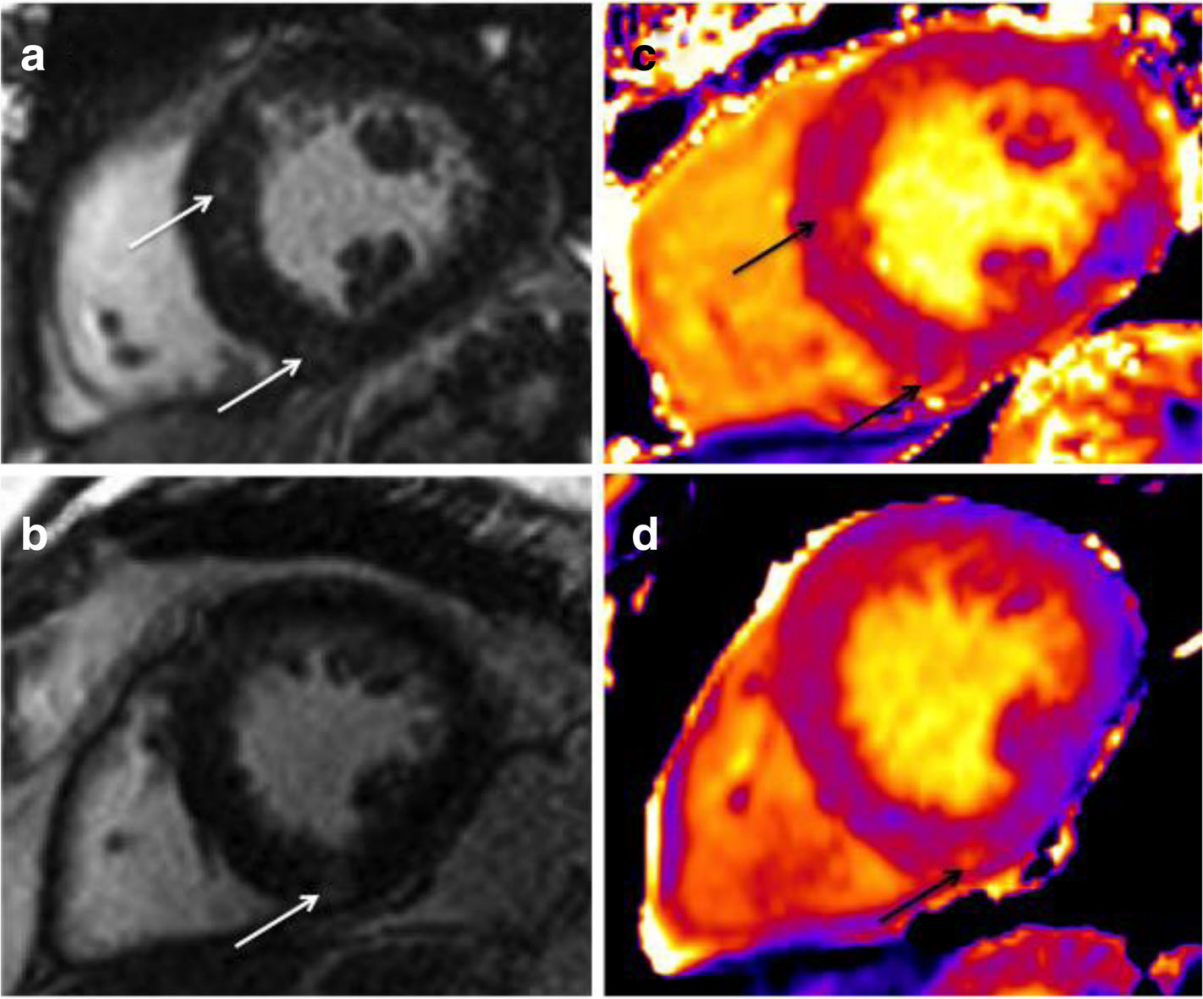
**a** Mid-ventricular LGE image of an AS patient with LGE scored 1 (diffuse subtle enhancement). Enhancement seen at RV inferior insertion point and septal mid-wall (arrows). **b** Mid-ventricular LGE image of AS patient scored 2 (strong and discrete enhancement). Discrete enhancement seen at RV inferior insertion point (arrow). **c** Mid-ventricular native T1 map of the same patient shown in Fig. ‘1a’. Areas of discretely increased signal visible in the same distribution as enhancing areas on LGE image (arrows) **d** Mid-ventricular native T1 map of the same patient shown in Fig. ‘1b’. Discrete area of increased native T1 signal clearly visible at RV insertion point (arrow)

Native T1 maps and LGE images were assessed separately, offline, two-weeks apart by blinded observers. For patients with AS, mid-ventricular LGE images were assessed by two, blinded observers (AS, GPM) for the presence of late gadolinium enhancement. Strongly enhancing areas were scored ‘2’, and weakly enhancing areas were scored ‘1’ (Fig. 1). Corresponding mid-ventricular native T1 maps of AS patients were assessed (separately) for areas of discretely increased signal likely to represent scar fibrosis (MGB, GPM). The locations of areas defined as scarring by the two techniques were compared. For haemodialysis patients, two blinded observers (MGB, GPM) assessed the mid-ventricular native T1 maps for areas of discretely increased signal likely to represent myocardial scar.

Image quality was assessed as being excellent, good, acceptable or poor.

### Quantitative image analysis

All scans were analysed offline by a single, blinded observer (MGB).

#### Native T1 mapping

Mid-ventricular native T1 parametric maps derived from MOCO MOLLI images were used for native T1 analysis due to superior intra- and inter-observer variability compared to analysing the individual image series [37]. Circumferential native T1 values of mid-ventricular slices were analysed as previously described using the native T1 analysis module of the software package CMR^42^ (Circle, Cardiovascular Imaging, Calgary, Alberta, Canada) [30].

##### Native T1 thresholds to assess extent of MF in patients with AS

Native T1 thresholds were systematically tested by deriving absolute values 2 to 9 standard deviations above the normal range for the 3 T CMR scanner (1083 ± 33 ms) [30]. This gave absolute thresholds of 1149 ms, 1182 ms, 1215 ms, 1248 ms, 1282 ms, 1314 ms, 1347 ms and 1380 ms.

One further technique was tested which set an individual native T1 threshold for each patient. This individual threshold was set by adding the standard deviation (SD) of the mean T1 in the region of interest thought to represent scar to the mean circumferential native T1 for the patient (T11SD).

**Fig. 2:**
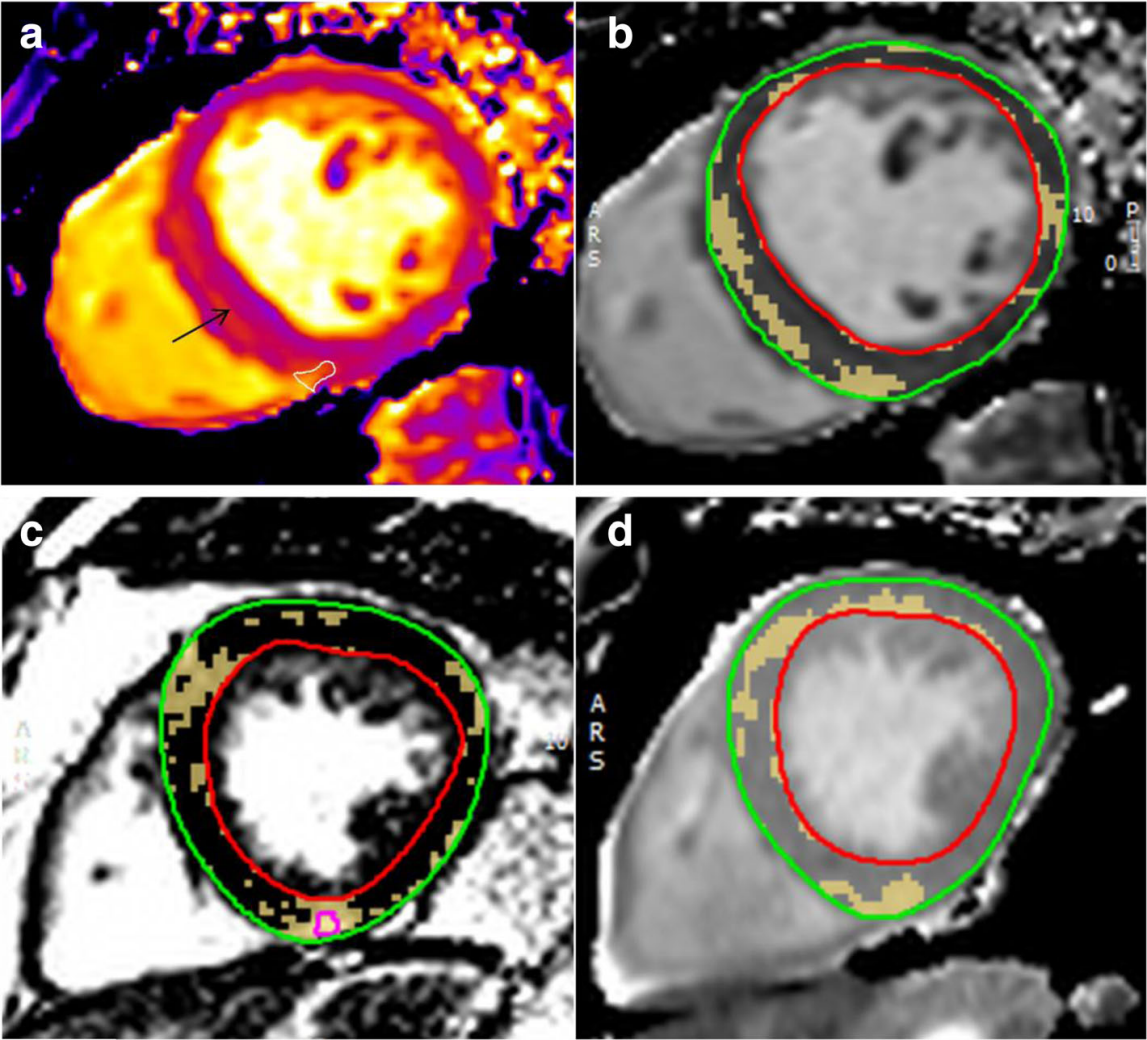
**a**: Native T1 map of a haemodialysis patient. Region of interested (white) defining visual area of greatest signal increase at right ventricular inferior insertion point. Black arrow shows septal mid-wall discretely increased native T1 signal **b**: The same Native T1 in CMR^42^ tissue characterization module set T1 threshold defined by the global native T1 for the patient plus the standard deviation of the region of interest circled as the area of highest signal increase (defined in Argus in 1A) (T11SD technique). **c**: Mid-ventricular late gadolinium enhanced image of an aortic stenosis patient analysed using full-width half-maximum in the CMR-42 tissue characterization module. **d**: Corresponding native T1 map of the same patient with aortic stenosis analysed with T11SD technique

The following techniques were used to derive native T1 values for the above calculations:

1. Native T1 parametric maps were inspected in Argus Viewer (Siemens Healthcare, Erlangen, Germany). On T1 maps where an area of discretely increased signal intensity could be seen, a region of interest was drawn around the area of highest signal and the mean and standard deviation of the T1 for that region was recorded (Fig. 2a).
2. Native T1 maps were then loaded into the LGE-tissue characterization module of the software package CMR42 (Circle, Cardiovascular Imaging, Calgary, Alberta, Canada). Epicardial and endocardial contours were drawn to define the myocardium. The absolute T1 signal threshold being tested (as above) was applied manually to give the percentage of myocardium within that signal intensity. Thresholds for the T11SD technique were derived and applied individually for each patient (Fig. 2b).

#### Late gadolinium enhanced images

Corresponding, mid-ventricular LGE images were analysed using software package CMR^42^. Epicardial and endocardial contours were applied and analysed using FWHM and the 3SD technique to generate a percentage area of fibrosis burden as previously described [17, 38]. The Full width half-maximum technique defines the enhanced area by using 50% of the maximum signal found within the enhanced area (Fig. 1c). The 3SD technique defines enhanced areas as 3 standard deviations above remote (normal) myocardium.

### Statistical analysis

Statistical analysis was undertaken using SPSS-22 software (Statistical Package for the Social Sciences, Chicago, IL, USA) and Graphpad Prism version 6.04 (GraphPad Software, Inc., La Jolla, CA, USA). Normality was assessed using the Shapiro–Wilk test, histograms, and Q–Q plots. Parametric data are expressed as mean ± standard deviation and non-parametric data are expressed as median (interquartile range). Chi-squared tests and Fishers exact tests were used to assess for differences between categorical variables and are expressed as ‘count’ (%). Continuous variables were compared by paired or independent t-tests and Mann-Whitney U tests. Agreement between the native T1 signal thresholding techniques and LGE analysis with FWHM to assess extent of fibrosis was assessed with intra-class correlation coefficient (ICC) and Bland-Altman analysis. Correlations between variables were assessed using Pearson’s and Spearman’s-rank analysis for normally and non-normally distributed data respectively. Inter-observer and intra-observer variability were assessed using ICC and Bland-Altman analysis. Repeatability was considered ‘excellent’ for ICC > 0.9, ‘good’ for ICC between 0.8–0.89, moderate for ICC between 0.5–0.79 and poor for < 0.5. A two-tailed *p*-value < 0.05 was considered statistically significant.

### Assessment of extent of MF in haemodialysis patients

Any native T1 thresholding technique that showed a significant correlation with FWHM or 3SD in AS patients was used to assess extent of MF in haemodialysis patients. Inter and intra-observer variability of native T1 mapping thresholding techniques were conducted on the native T1 maps of 10 haemodialysis patients. Inter-observer variability was assessed by comparing the results of two blinded observers and intra-observer variability was assessed by comparing the results of 1 blinded observer who undertook re-analysis of 10 scans at random 4 weeks after initial analysis.

## Results

### Participant characteristics

Demographic, clinical, biochemical and additional CMR data are shown in Table 1. There were no differences in gender or BMI between the study groups. The AS patients were older than haemodialysis patients. There were no significant associations between age and native T1 in haemodialysis or AS patients (*r* = 0.22 and *r* = 0.21 respectively). Systolic blood pressure was significantly lower in haemodialysis patients without areas of discretely increased signal on native T1 maps compared to AS patients and to haemodialysis patients with areas of discretely increased signal on native T1 maps.

**Table 1:** Demographic details of Aortic Stenosis and haemodialysis patients

Variable	Aortic Stenosis patients (areas of discrete signal increase on native T1) (*n* = 25)	Haemodialysis patients areas of discrete signal increase on native T1) (n = 25)	Haemodialysis patients (no areas of discrete signal increase on native T1) (*n* = 19)
Age (years)	66.9 ± 13.7	58.1 ± 16.5	49.9 ± 16.9
Male (*n*, %)	18 (72)	18 (72)	16 (84)
BMI (kg/m^2^)	29.4 ± 4.4	27.8 ± 6.8	27.9 ± 6.4
Dialysis Vintage (months)	–	35.4 ± 25.8	26.9 ± 21.6
Haemoglobin (g/L)	14.5 ± 1.2	11.4 ± 1.6	10.4 ± 1.6
HbA1c (%)	5.9 ± 0.9	5.4 ± 1.8	5.4 ± 0.6
SBP (mmHg)	158.8 ± 21.4	152.6 ± 22.5	127.6 ± 25.8
DBP (mmHg)	78.3 ± 9.6	79.5 ± 10.5	72.5 ± 19.1
HR (bpm)	70.1 ± 10	72.8 ± 12.5	78.1 ± 11.1
Past medical and drug history			
Hypertension (*n*, %)	18 (72)	21 (84)	11 (58)
Diabetes (*n*, %)	4 (16)	7 (28)	4 (21)
CAD (*n*, %)	11 (44)	9 (36)	4 (21)
Prev MI (*n*, %)	3 (12)	5 (20)	1 (5)
ACEi (*n*, %)	7 (28)	2 (8)	1 (5)
ARB (*n*, %)	4 (16)	3 (12)	1 (5)
Diuretic (*n*, %)	7 (28)	3 (12)	4 (21)
Beta Blocker (*n*, %)	10 (40)	12 (48)	6 (32)
Statin (*n*, %)	17 (68)	14 (56)	5 (26)
Calcium Channel Blocker (*n*, %)	11 (44)	11 (44)	5 (26)
Number of antihypertensives	1.6 ± 1.2	1.4 ± 0.9	1.3 ± 1.1
Left Ventricular mass and volumes (CMR)			
LVEDV (ml)	190.3 ± 43.4	198.8 ± 54.3	160.1 ± 48
LVEF (%)	56.5 ± 6.5	52.2 ± 6.4	50.5 ± 6.2
LVM (g)	135.4 ± 42.4	116.1 ± 29.4	104.1 ± 45.3
LVM/LVEDV (g/ml)	0.71 ± 0.1	0.59 ± 0.1	0.62 ± 0.1

Mean values with standard deviation expressed as n ± SD. N, % = Chi-squared. bpm, beats per minute; *ACEi* angiotensin converting enzyme inhibitor, *ARB* angiotensin receptor blocker, *CAD* coronary artery disease, *CMR* cardiac MRI, *DBP* diastolic blood pressure, *HR* heart rate, *HTN* hypertension, *LVEDV* left ventricular end-diastolic volume, *LVEF* left ventricular ejection fraction, *LVMi* left ventricular mass index, *MI* myocardial infarction, *SBP* systolic blood pressure

### Visual assessment and image quality on native T1 maps and LGE images

**Fig. 3:**
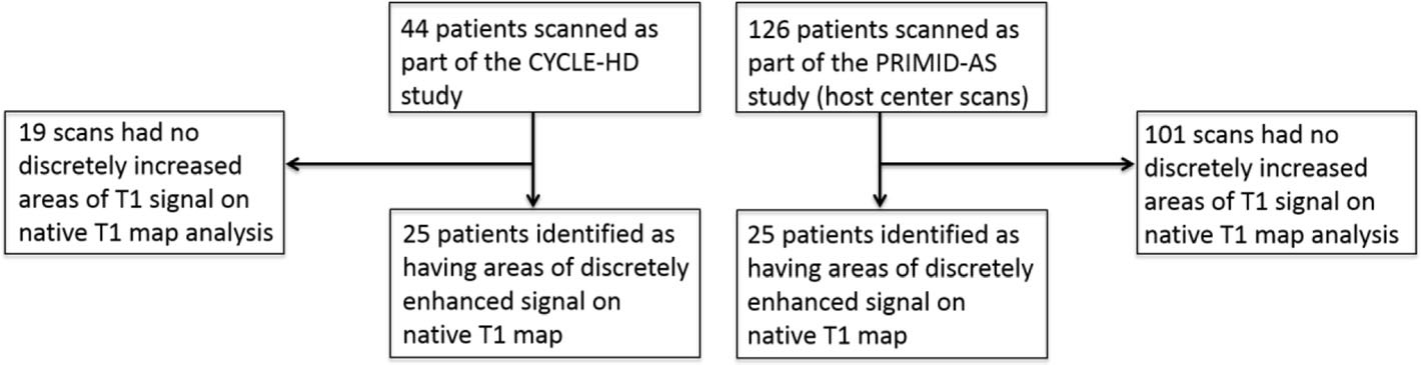
Flow diagram of patients included in study and numbers of patients with areas of discretely increased signal on native T1 maps

Twenty-five out of 126 AS patients had areas of enhancement in a mid-ventricular LGE image (Fig. 3). All of these patients had a corresponding native T1 map at the same slice position that were assessed separately for areas of discretely increased signal. Fifteen of the LGE images were scored ‘excellent’, 8 were scored ‘good’ and 2 were scored as acceptable. Similarly, 14 of the native T1 maps were scored as ‘excellent’ and 11 were scored as ‘good’. In patients with AS, 12 areas of enhancement on LGE images were scored as ‘1’ and 13 were scored as ‘2’. Twenty six areas of enhancement were identified on LGE images, with 25 corresponding areas of discretely increased native T1 signal identified on corresponding mid-ventricular native T1 maps (Table 2).

**Table 2:** Location of signal enhancement on LGE images in AS patients and of discretely increased signal on native T1 maps in AS and haemodialysis patients

Area of enhancement/increased signal intensity	LGE images in AS patients (number)	Native T1 maps in AS patients (number)	Native T1 map in HD patients (number)
Inferior insertion point	17	17	17
Anterior insertion point	3	3	3
Septal mid-wall	1	1	14
Infero-lateral mid-wall	3	2	1
Lateral mid-wall	2	2	0
Inferior wall	0	0	4

*HD* haemodialysis, *AS* aortic stenosis

The mid-ventricular native T1 maps of 44 haemodialysis patients were reviewed and areas of discretely enhanced signal were identified in 25 patients (Fig. 3). For haemodialysis patients, 24 of the native T1 maps were scored as ‘excellent’ and 20 were scored as ‘good’. Locations of areas of discretely increased signal are shown in Table 2.

### Global and regional native T1 in AS and haemodialysis patients

**Fig. 4:**
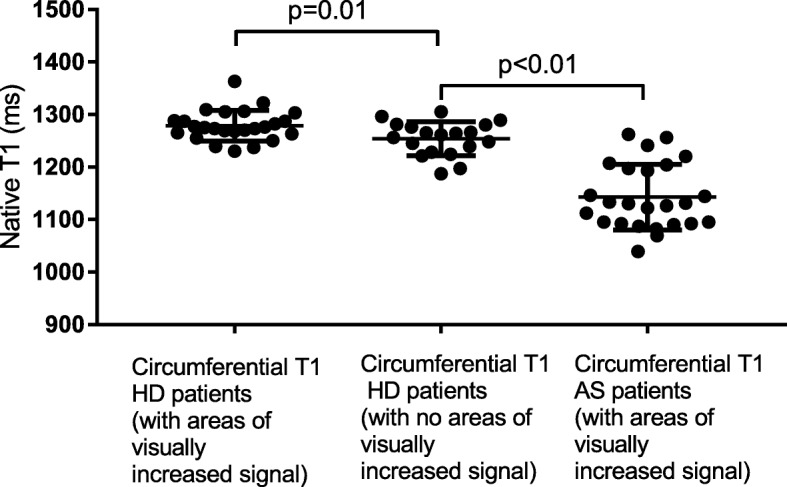
Circumferential native T1 in haemodialysis patients with areas of visually increased signal, haemodialysis patients without areas of visually increased signal and aortic stenosis patients with areas of visually increased signal

Global native T1 values and native T1 values within discrete regions of interest thought to represent MF for AS and haemodialysis patients are shown in Table 3. Circumferential native T1 values were significantly lower in AS patients (*n* = 25), compared to patients on haemodialysis patients (*n* = 44) (1143 ms ± 12.49 vs 1268.2 ± 32.5, *P* < 0.001) (Fig. 4). Patients on haemodialysis who had areas of discretely increased signal on T1 mapping (*n* = 25) had significantly higher circumferential native T1 than those without (*n* = 19) (1279 ms ± 5.8 vs 1254 ms ± 7.4, *P* = 0.01).

**Table 3:** Global and regional native T1 values in patients with aortic stenosis and those on haemodialysis

Patient Group	Circumferential Native T1 (ms)	Native T1 in region of interest thought to represent scar (ms)
Patients with aortic stenosis (*n* = 126)	1125.8 ± 57.5	–
Patients with aortic stenosis with areas of discretely increased signal on native T1 (*n* = 25)	1142.6 ± 62.4	1275.8 ± 102.4
Patients on haemodialysis (*n* = 44)	1268.2 ± 32.5	–
Patients on haemodialysis with areas of discretely increased signal on native T1 (*n* = 25)	1278.9 ± 28.9	1390.3 ± 43.3

**Fig. 5:**
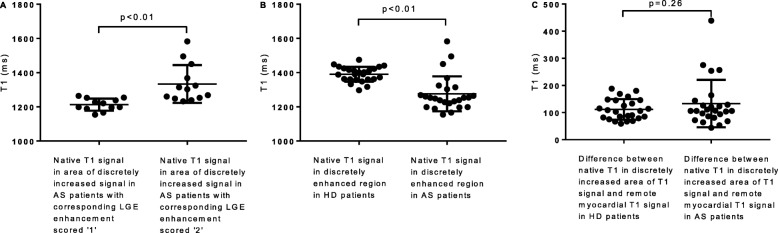
**a**: Comparison of corresponding areas of discretely increased signal on native T1 in aortic stenosis patients with LGE images scored ‘1’ and scored ‘2’. **b**: Native T1 signal within region of discrete signal increase in haemodialysis patients compared to native T1 signal within region of discrete signal increase in aortic stenosis patients. **c**: Difference in native T1 between remote myocardium and myocardium within areas of discretely increased signal between haemodialysis and aortic stenosis patients. ms, milliseconds; HD, haemodialysis,; AS, aortic stenosis; LGE, late gadolinium enhancement

For AS patients, regions of interest on native T1 that corresponded to an area of LGE scored as a ‘1’ (*n* = 12) were significantly lower than regions of interest on native T1 that corresponded to an area of LGE that was scored as a ‘2’ (*n* = 13) (1213 ms ± 10.2 vs 1334 ms ± 30.7, *P* < 0.01) (Fig. 5). The areas of discretely enhanced native T1 signal, defined by regions of interest, were significantly higher in haemodialysis patients (*n* = 25) than in patients with AS (n = 25) (1390 ± 8.7 vs 1276 ms ± 20.5, P < 0.01) and significantly higher than the regions of interest that corresponded with LGE scored ‘2’ (*n* = 13) (1390 ± 8.7 vs 1334 ± 30.72, *P* = 0.03) (Fig. 3). The difference in native T1 signal in the region of increased signal intensity and that of remote myocardium was not significantly different between haemodialysis patients and patients with AS (111.4 ms ± 7.6 vs 133.2 ms ± 17.5, *P* = 0.26) (Fig. 4).

Nine patients on haemodialysis had a previous history of coronary artery disease and 16 patients did not. There were no differences in the global native T1 times or the T1 times with regions of greater signal intensity for patients with a prior history of coronary artery disease and those without (global native T1 1279.2 ± 30.6 vs 1278.7 ± 28.9, *p* = 0.37 and highest regional native T1 1366.0 ± 36.5 vs 1404.0 ± 41.6, *p* = 0.35).

### Native T1 thresholding techniques compared to FWHM in AS patients

#### Absolute native signal thresholds

None of the absolute signal thresholding techniques used to define extent of MF based on an absolute value derived from standard deviations above our normal range for native T1 showed significant correlation or agreement with extent of MF defined by either FWHM or 3SD (Table 4).

**Table 4:** Agreement between native T1 thresholding techniques and FWHM to define extent of myocardial fibrosis

Native T1 threshold/technique (standard deviation above normal for native T1)	Extent of fibrosis defined by FWHM (%)	Extent of fibrosis defined by technique (%)	Correlation between FWHM and T1 technique (r)	Agreement between extent of MF defined by T1 thresholding technique and FWHM (ICC)
1149 ms (2SD)	14.38 ± 1.5	44.6 ± 33.7*	0.07	0.06
1182 ms (3SD)	14.38 ± 1.5	32.8 ± 32.6*	0.1	0.07
1215 ms (4SD)	14.38 ± 1.5	22.9 ± 26.3	0.11	0.11
1248 ms (5SD)	14.38 ± 1.5	13.3 ± 17.3	0.15	0.2
1281 ms (6SD)	14.38 ± 1.5	6.7 ± 10*	0.21	0.26
1314 ms (7SD)	14.38 ± 1.5	3.5 ± 5*	0.23	0.16
1357 ms (8SD)	14.38 ± 1.5	1.8 ± 2.3*	0.17	0.05
1380 ms (9SD)	14.38 ± 1.5	1 ± 1.3*	0.02	0.03
T11SD	14.38 ± 1.5	18.24% ± 1.4*	0.55†	0.64††
Native T1 threshold/technique (standard deviation above normal for native T1)	Extent of fibrosis defined by LGE 3SD (%)	Extent of fibrosis defined by technique (%)	Correlation between LGE 3SD and T1 technique (r)	Agreement between extent of MF defined by T1 thresholding technique and LGE 3SD (ICC)
1149 ms (2SD)	21.1 ± 12.1	44.6 ± 33.7*	0.03	0.04
1182 ms (3SD)	21.1 ± 12.1	32.8 ± 32.6*	0.075	0.1
1215 ms (4SD)	21.1 ± 12.1	22.9 ± 26.3	0.11	0.18
1248 ms (5SD)	21.1 ± 12.1	13.3 ± 17.3	0.09	0.18
1281 ms (6SD)	21.1 ± 12.1	6.7 ± 10*	0.08	0.16
1314 ms (7SD)	21.1 ± 12.1	3.5 ± 5*	0.11	0.17
1357 ms (8SD)	21.1 ± 12.1	1.8 ± 2.3*	0.19	0.15
1380 ms (9SD)	21.1 ± 12.1	1 ± 1.3*	0.26	0.12
T11SD	21.1 ± 12.1	18.24% ± 1.4	0.1	0.2

Abbreviations: *FWHM* Full width half maximum, *ICC* intra-class correlation coefficient, *ms* milliseconds, *SD* Standard deviation. *denotes significant difference between native T1 threshold technique for extent of fibrosis and extent of fibrosis defined by FWHM (*P* < 0.05). †denotes significant correlation between FWHM and native T1 thresholding technique (*P* < 0.05). ††denotes significant agreement between FWHM and native T1 thresholding technique (*P* < 0.05)

#### T11SD

The native T1 threshold derived using T11SD was derived individually for each patient and was different in each case. There was no correlation or agreement between mean percentage area defined as MF by T11SD and 3SD. The mean percentage area defined as ‘MF’ by T11SD was higher than the mean area identified as scar using FWHM (18.24% ± 1.4 vs 14.38 ± 1.5, *P* = 0.043) (Fig. 4), but there was a moderate correlation and agreement between the two techniques (*r* = 0.55, *P* < 0.01, ICC = 0.64, P < 0.01). Bland-Altman analysis suggested the T11SD systematically overestimated extent of scar burden by 4.3% compared to FWHM (Bias 4.3 (SD of bias 6.8), limits of agreement − 9.1 - 17.7).

### Extent of myocardial fibrosis defined by T11SD in AS patients and HD patients

**Fig. 6:**
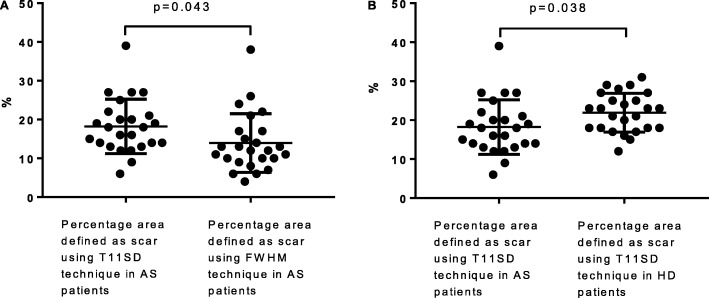
**a**: Percentage area defined as scar by T11SD on native T1 mapping compared to percentage area defined as scar by FWHM on LGE images in AS patients. **b**: Comparison of extent of scar defined by T11SD between aortic stenosis patients and patients on haemodialysis. AS, aortic stenosis; HD, haemodialysis

Mean percentage identified as ‘MF’ using T11SD was significantly greater in haemodialysis patients (*n* = 25) than patients with AS (n = 25) (21.92% ± 1 vs 18.24% ± 1.4, *P* = 0.038) (Fig. 6).

### Inter and intra-observer variability of T11SD in HD patients

Inter- and intra-observer variability for 10 haemodialysis patients are shown in Table 5. Bland-Altman analysis revealed no evidence of systematic bias, and all data points were within 95% confidence intervals (data not shown).

**Table 5:** Inter and intra-observer variability for T11SD analysis in haemodialysis patients

Parameter	Study 1	Study 2	ICC	BIAS ± SD Difference	BA Limits of Agreement
Inter-observer variability					
T11SD	22.32 ± 7	22.6 ± 7.8	0.87 (p < 0.01)	0.5 ± 5.5	−10.2 – 11.21
Intra-observer variability					
T11SD	22.32 ± 7	23.1 ± 8.1	0.96 (p < 0.01)	−0.8 ± 3.1	−6.8 – 5.2

*BA* Bland-Altman, *SD* standard deviation

## Discussion

Although we have shown that areas of discretely increased signal on native T1 mapping are visible and appear to correspond to areas LGE, we were not able to define an absolute native T1 signal threshold to define the extent of MF compared to FWHM or 3SD in AS patients. The areas of myocardium identified as having discretely increased signal on native T1 mapping in patients with AS were mostly located at the right ventricular inferior insertion point and matched well to enhanced areas on LGE images. The right ventricular insertion point was also a common area to show discretely increased native T1 signal in haemodialysis patients. Additionally in haemodialysis patients there were a large number of areas of discretely increased native T1 signal in the septum in a mid-wall pattern similar to that described by Mark et al. in the largest study using LGE in haemodialysis patients, before the risks of nephrogenic systemic fibrosis became apparent [39].

The reasons for not being able to define an absolute native T1 threshold to define extent of MF are not clear. Commentators have suggested that it should be possible to define absolute thresholds for identification of MF using T1 mapping, but acknowledge that there will be significant overlap in values between diseases [40]. Our observation that quantitative native T1 analysis differentiated between LGE scored ‘1’ and LGE scored ‘2’ in AS patients supports the idea that the technique is able to differentiate between levels of disease on a spectrum from DMF to replacement MF for discrete disease populations. Indeed studies have shown that native T1 threshold-based analysis for characterization of replacement MF in chronic myocardial infarction are excellent [41]. An alternative explanation for not being able to establish an absolute value to define the total extent of MF may be due to additional influences on myocardial native T1 beyond levels of MF. The observation that differences between native T1 in areas of discretely increased signal and remote myocardium in haemodialysis and AS patients were similar (111.4 ± 7.6 ms and 133.2 ± 17.5 ms) despite significantly higher native T1 values in haemodialysis patients suggests that global differences in background myocardium may account for the absolute differences in native T1 values in areas of discretely increased signal. These differences in background myocardium may be related to many factors, including levels of inflammation, oedema or amyloid infiltration (known to lengthen T1), or iron or triglyceride deposition (known to shorten T1) as well as absolute levels of MF. Indeed, factors unrelated to myocardial tissue such as haemoglobin concentration/haematocrit, blood vessels or capillary density may also introduce systematic differences between disease populations [17]. Theoretically the T11SD technique we have described accounts for differences in background myocardium on an individual basis. However, the technique is still reliant on visually identifying an area of discrete signal increase thought to be replacement MF (and is consequently subject to the same limitations of underestimating extent of MF as LGE SI techniques), but thereafter the individual threshold is set using the patient’s own native T1 values. For patients with high levels of myocardial inflammation or potentially at risk of myocardial oedema (such as haemodialysis patients), these influences on T1 are accounted for in the calculation of extent of MF. The fact that we were able to demonstrate moderate agreement between extent of MF defined by FWHM and T11SD supports this theory, although as the technique did not correlated with 3SD confirmatory studies, validation against histology and refining of the technique are required. This finding may have implications that extend beyond use in haemodialysis patients, as non-enhanced scans should now be considered (when suitable) for patients with gadolinium brain deposits.

The absolute differences between native T1 in areas of discretely increased signal and remote myocardium between haemodialysis and AS patients (111.4 ms and 133.2 ms) represent a 9.6 and 10.4% increase in native T1 signal above background myocardium. These thresholds are lower than the 21% difference described in a study that examined the use of native T1 mapping at defining scarring following myocardial infarction at 3 T [41]. This is unsurprising for two reasons. Firstly, the density of fibrosis following myocardial infarction is likely to be greater, and often transmural, causing relatively higher native T1 times. Secondly, the background (remote) myocardium in patients following acute myocardial infarction is likely to have less DMF than expected in patients with both AS and those on haemodialysis. Consequently, remote myocardial native T1 is likely to be significantly lower in patients following acute myocardial infarction, and the difference between remote myocardium and scarred myocardium greater.

The inter- and intra-observer variability for T11SD in haemodialysis patients were excellent, and equivalent to assessment with FWHM [19]. This is essential as it is the repeatability of LGE SI techniques, rather than their absolute accuracy, that have made them useful biomarkers of extent of MF in multi-centre clinical trial work. The T11SD technique is experimental, however, and theoretically is subject to the same limitations as LGE for identifying DMF and therefore should be used as hypothesis generating only.

Circumferential native T1 values were significantly higher in haemodialysis patients than AS patients. This difference in myocardial characteristics may be due to higher levels of DMF; indeed histological studies support this hypothesis as non-ischaemic DMF is a prominent feature of cardiomyopathy in haemodialysis patients [7, 8]. However, due to lack of histological validation of native T1 mapping in haemodialysis patients, it remains possible that this increased native T1 signal may be caused by increased myocardial water content, either from myocardial oedema, chronic inflammation or haemoglobin concentration rather than MF. We have previously shown the reproducibility of circumferential native T1 mapping to be excellent, and unrelated to changes in markers of fluid status [42]. Confirming our findings, a recent study, in which intra-dialytic CMR was performed, showed that haemodialysis and ultrafiltration had no acute effect on native T1 value [43]. Together, these studies make it highly unlikely that myocardial oedema from fluid overload contributes to elevated native T1 in haemodialysis patients. It is possible that elevated circumferential native T1 in haemodialysis is due to a combination of high levels of DMF and low grade myocardial inflammation or systematic differences within this population such as anaemia.

### Limitations

This is a single centre study with a relatively small sample size, though larger than previous similar studies [41, 44]. Whilst the technique of T11SD correlates with FWHM for defining extent of MF, it did not correlate with 3SD, which is arguably a more accurate method of defining extent of MF in AS patients [17]. We do not have histological validation of this technique, or histological validation of native T1 mapping for the definition of MF in patients with renal disease. The haemodialysis group without discretely enhanced areas on native T1 map had significantly lower systolic blood pressure, were younger and were of a shorter dialysis vintage than haemodialysis patients without areas of discretely increased signal on native T1 maps. Native T1 values are known to be higher in patients with essential hypertension than controls [45] and whilst we found no significant association between blood pressure and native T1 amongst haemodialysis patients we did observe a trend. Increased blood pressure is likely to play a central role in the development of DMF and replacement fibrosis in haemodialysis patients as is time on dialysis and age, and these results should be investigated further in larger studies. As expected, haemoglobin was significantly lower in haemodialysis patients compared to AS patients. Although values for haemoglobin were in the target range for dialysis patients, (as previously reported [30]), this may contribute to the higher native T1 values we see in haemodialysis patients. We did not, however, find any relationship between haemoglobin and native T1 time in haemodialysis patients. We did not observe any difference between global native T1 times or T1 times in regions of greatest signal intensity between the haemodialysis patients with and without coronary artery disease. This might suggest processes related to the effects of CKD and dialysis lead to the areas native T1 and the global increases in native T1 that we observed, but because there was no angiographic component to this study we cannot be sure that patients without a history of coronary artery disease did not have occult, undiagnosed disease. In this study LGE images were used as the reference values for extent of MF. LGE is the gold-standard for the identification and definition of replacement fibrosis, but extracellular volume measurement (ECV) may be a better non-invasive reference standard for quantification of extent of MF, as ECV (like native T1 mapping) is a continuous variable that allows quantification of ECV expansion in LGE negative areas.

## Conclusions

Visual identification of T1 maps was the most effective technique at identifying areas of LGE in AS patients which is considered our standard for MF evaluation. We were unable to identify an absolute native T1 threshold to define extent of MF. This work supports the position of the Society for Cardiovascular Magnetic Resonance and CMR Working Group of the European Society of Cardiology that researchers must continue to phenotype native T1 values across diseases, and define normal and pathological ranges on individual scanners [46]. Histological studies are needed to assess the ability of native T1 mapping and native T1 map signal thresholding techniques to define extent of MF and replacement fibrosis in patients with renal disease.

## Abbreviations

3SD: 3 standard deviations
AS: Aortic stenosis
CKD: Chronic kidney disease
CMR: Cardiac magnetic resonance imaging
DMF: Diffuse myocardial fibrosis
ESRD: End-stage renal disease
FWHM: Full width half-maximum
ICC: Intra-class correlation coefficient
LGE: Late gadolinium enhancement
LV: Left ventricle
MF: Myocardial fibrosis
MOCO: Motion corrected
MOLLI: Modified look-locker inversion recovery
MRI: Magnetic resonance imaging
SI: Signal intensity
T11SD: Native T1 threshold technique calculated by applying the standard deviation of a region of interest thought to represent scarring on native T1 above the mean circumferential native T1 for the same patient
